# Multiphotonic Ablation and Electro-Capacitive Effects Exhibited by *Candida albicans* Biofilms

**DOI:** 10.3390/bioengineering11040333

**Published:** 2024-03-28

**Authors:** Jose Alberto Arano-Martinez, José Alejandro Hernández-Benítez, Hilario Martines-Arano, Aída Verónica Rodríguez-Tovar, Martin Trejo-Valdez, Blanca Estela García-Pérez, Carlos Torres-Torres

**Affiliations:** 1 Sección de Estudios de Posgrado e Investigación, Escuela Superior de Ingeniería Mecánica y Eléctrica Unidad Zacatenco, Instituto Politécnico Nacional, Ciudad de México 07738, Mexico; 2 Departamento de Microbiología, Escuela Nacional de Ciencias Biológicas, Instituto Politécnico Nacional, Ciudad de México 11340, Mexico; 3 Escuela Superior Tepeji del Río, Universidad Autónoma del Estado de Hidalgo, Tepeji del Río de Ocampo, Hidalgo 42860, Mexico; 4 Escuela Superior de Ingeniería Química e Industrias Extractivas, Instituto Politécnico Nacional, Ciudad de México 07738, Mexico

**Keywords:** optical ablation, optical absorbance, *Candida albicans*, electrical capacitance, nonlinear optical absorption, Nd:YAG laser

## Abstract

This work reports the modification in the homogeneity of ablation effects with the assistance of nonlinear optical phenomena exhibited by *C. albicans* ATCC 10231, forming a biofilm. Equivalent optical energies with different levels of intensity were irradiated in comparative samples, and significant changes were observed. Nanosecond pulses provided by an Nd:YAG laser system at a 532 nm wavelength in a single-beam experiment were employed to explore the photodamage and the nonlinear optical transmittance. A nonlinear optical absorption coefficient −2 × 10^−6^ cm/W was measured in the samples studied. It is reported that multiphotonic interactions can promote more symmetric optical damage derived by faster changes in the evolution of fractional photoenergy transference. The electrochemical response of the sample was studied to further investigate the electronic dynamics dependent on electrical frequency, and an electro-capacitive behavior in the sample was identified. Fractional differential calculations were proposed to describe the thermal transport induced by nanosecond pulses in the fungi media. These results highlight the nonlinear optical effects to be considered as a base for developing photothermally activated phototechnology and high-precision photodamage in biological systems.

## 1. Introduction

*Candida albicans* is a common fungus in the human microbiota. This fungus is an opportunistic pathogen that infects immunocompromised people. The physical properties of *C. albicans* represent critical aspects for developing therapeutic strategies for infections caused by this yeast. In recent years, many scientists have studied how to destroy common fungi with different techniques, and particular examples are associated with photothermal methods [1]. Photothermal procedures use heat induced by light to successfully remove the fungi without the use of harsh chemicals or medications [2]. In this topic, the term “photothermal effects” is related to the material’s capacity to capture light energy and transform it into heat, which can be used to kill cells or microorganisms [3]. It has been reported that *C. albicans* exposed to light results in photothermal reactions that effectively kill the yeast [4].

Photothermal investigations offer a good alternative to mitigate fungal infections in humans, offering a minimally invasive and highly efficient therapeutic option compared to existing options [5]. Although photothermal effects are related to the interaction between light and matter, it is important to understand that linear and nonlinear optical effects are assisted by different physical mechanisms responsible for biological functions. When light encounters materials, optical phenomena such as fluorescence, absorption, reflection, and refraction occur [6]. In contrast, nonlinear effects involve the simultaneous absorption of several photons by a molecule or group of molecules [7].

It has been demonstrated that phototherapy [8] and antimicrobial photodynamic therapy [9] are an effective strategy against *C. albicans* infection. Moreover, the assistance of optical nonlinearities promoted by femtosecond lasers seems to promise positive results; however, it is necessary to optimize the parameters of irradiation to improve the elimination of bacteria [10] and improve the quality of the photoinduced effects [11].

Ultrafast multiphotonics may cause photodamage, photobleaching, photopolymerization, and photoactivation, which are all examples of biological reactions with or without the presence of heat. Optical ablation in *C. albicans* can be targeted by selecting specific cells or compounds unique to the fungus [12]. For example, some lasers could localize the cell wall of *C. albicans* samples, which comprises elements such as chitin and glycans [13]. Through this targeted approach, the laser efficiently kills *C. albicans* cells and simultaneously preserves the integrity of healthy human cells by exclusively damaging the fungal cell wall.

Laser light can be employed in optical ablation methods to destroy or eliminate certain cells or tissues [14]. *C. albicans* can cause diverse infections determined by the role of specific proteins during growth and survival [15]. The heat response of *C. albicans* to laser ablation is responsible for fundamental aspects of photoinduced damage [16]. Variations in the room temperature can have a substantial influence on the health and behavior of the microorganism sample [17]. Several scientific reports have suggested that biological tissue may experience temperature changes because of laser irradiation and these alterations affect the morphology, viability, and metabolic processes of the yeast [18].

It is worth mentioning that in order to describe photothermal processes, fractional calculus introduces non-integer differentiation and integration tools [19]. This tool provides the opportunity for a more versatile and effective modeling approach to interpret more complicated interactions. Incorporating fractional calculus in the mathematical description of photothermal transport becomes particularly essential when interpreting temperature change behavior. Fractional calculus becomes useful in describing the behavior of materials in physical processes [20]. The advantages of fractional calculus are also attractive for the simulation of various phenomena, such as the dynamics of excitons in the diffusion of charge carriers in optical materials [21].

An important part of the progress of the techniques to reduce *C. albicans* infections has considered the assistance of laser light irradiation [22]. It has been shown a notable decrease in the formation of *C. albicans* biofilms using a photopolymerized film in the presence of light [23]. Furthermore, innovative approaches, such as IgY photoantimicrobial-targeted treatments, have been employed in near-infrared photoimmunotherapy to counteract *C. albicans* [24]. The efficiency of photodynamic inactivation using a 660 nm diode laser has been successfully developed to inactivate *C. albicans* [25]. Additionally, laser therapies involving Ti alloy surfaces have been employed to inhibit *C. albicans* biofilms and alter their cellular morphology [26].

It is worth noting that electrical and electromagnetic functions in biological systems represent an attractive topic of research that has been devoted to elucidating fundamental aspects of macromolecules in cells. It has been evidenced that the electric field can modify protein conformation; additionally, the electric field also can change the unfolding mechanism and modify the aggregation process before ablation to induce deformation and reorient the amorphous aggregates [27,28]. These effects together have a key impact on protein biological functionality, implying advantages in various disciplines where the protein aggregates are essentially important as pharmacology [29], food processing [30], materials science [31], and medicine [32]. Importantly, the direct use of an electric field could achieve microbial or enzymatic inactivation or electroporation of cellular structures [33], which represents an extraordinary advantage to the control of microorganism growth. In addition, moderate homogeneous electric fields can lead DNA molecules to compress and self-entangle [34], which yields perspectives in medical areas, such as gene therapy and DNA-based nanofabrication, using any kind of cell. Furthermore, the progress of laser technology for biological sciences envisions the use of multiphotonic interactions for developing high-precision tools for imaging, photodamage, and photothermally activated functions. Considering these facts, the study presented in this manuscript provides crucial details on the potential of laser-based techniques to remove *C. albicans* planktonic cultures and biofilms. The development of efficient treatments for *Candida albicans* infections, driven by nonlinear optic effects and described by fractional differential calculus, can be a base for future research.

*C. albicans* may be found in a variety of regions throughout the body, notably the throat, mouth, gut, and genital region [35]. However, the immune system regulates *C. albicans* growth. Certain conditions can weaken the immune system or modify the body’s natural balance, allowing the yeast to uncontrollably grow and cause infection [36]. A clear influence of fungi activity in a biological system can be provided by changes in electron transport and membrane permeability of the yeast [37]. With all these considerations, this work has been devoted to exploring the optical, thermal, and electrical characteristics of *C. albicans* biofilms. It highlights the potential of high-irradiance optical signals to improve selective laser ablation effects described by multiphotonics, electro-capacitive behavior, and fractional thermal transport.

## 2. Materials and Methods

### 2.1. Strain and Inoculum Preparation

*C. albicans* ATCC 10231 stored in YPD glycerol broth (1:1 *v*/*v*) at −80 °C was cultivated in YPD broth [1% yeast extract (MCD Lab, Tlalnepantla, Estado de México, Mexico), 2% peptone (MCD Lab, Tlalnepantla, Estado de México, Mexico), 2% dextrose (DIBICO^®^, Cuautitlán Izcalli, Estado de México, Mexico)]. For inoculum preparation, blastoconida were counted and adjusted at an inoculum concentration blastoconidia/mL in MOPS-buffered Roswell Park Memorial Institute 1640 medium (RPMI) (Gibco^®^, Thermo Fisher Scientific, Waltham, MA, USA) supplemented with 2% dextrose (DIBICO^®^, Cuautitlán Izcalli, Estado de México, Mexico).

### 2.2. In Vitro Biofilm Formation by C. albicans ATCC 10231

After inoculum adjustment, the biofilms were formed as previously described [38]. For brightfield microscopy, a vitro biofilm was placed over sterile glass coverslips in the bottom of a 12-well polystyrene plate and incubated at 37 °C for 24 h. After incubation, culture media were discarded, and the studied biofilm was washed twice with PBS 1X, coverslip, recovered, and observed in brightfield microscopy (Primo Star, Carl Zeiss, Jena, Germany). For epifluorescence microscopy, after the biofilm synthesis, the sample was washed with PBS 1X, fixed with 4% paraformaldehyde (Sigma-Aldrich, St. Louis, MO, USA), covered, and incubated with 1 mg/mL Concanavalin A (Sigma-Aldrich, St. Louis, MO, USA); after that, the sample was washed, and the coverslips were recovered and observed in an epifluorescence microscope (LSM Carl Zeiss, Jena, Germany) with a 490–519 nm filter.

For scanning electron microscopy (SEM) observations, yeast biofilms were prepared over 316 L steel discs at the bottom of a 12-well polystyrene plate. After incubation, the culture media were discarded, and the biofilm was washed with PBS 1X. The biofilms were fixed with 2.5% glutaraldehyde (Sigma-Aldrich, St. Louis, MO, USA) and post-fixed with 1% osmium tetroxide (Sigma-Aldrich, St. Louis, MO, USA); PBS 1X was used to rinse after every treatment. The samples were dehydrated by increasing concentrations of ethanol, dried by critical point with Hexamethyldisilazane (Electron Microscopy Sciences, Hatfield, PA, USA), and metalized for observation by SEM (Quanta 3D, FEG, FEI).

For optical, electrical, and thermal studies, *C. albicans* ATCC 10231 in vitro biofilms were set up with blastoconidia/mL over sterile glass coverslips at the bottom of a 12-well polystyrene plate and incubated at 37 °C for 24 h. After incubation, glass coverslips were recovered and exposed to different light shots to evaluate ablation effects.

### 2.3. Electrochemical Impedance Explorations in C. albicans ATCC 10231

Electrical impedance is a measurement of opposition to the flow of an electric current by media. It can be used to investigate the dynamic electronic effects exhibited by biological systems. The capacity impedance measures the ability of a system to retain electrical charge; an inductor’s impedance is directly proportional to frequency and a resistor’s impedance remains constant with frequency. The experiments in our sample were undertaken using a Autolab PGSTAT302, (Metrohm, Switzerland). We employed the Electrochemical Impedance Spectroscopy (EIS) technique to obtain electrical impedance parameters called electrolysis. We used a drop containing the *C. albicans* fungus on a DS 220 AT electrode (Metrohm, Switzerland) from the Metrohom brand.

A fundamental characteristic of electrical circuits that combines resistance and reactance is total impedance, indicated by the symbol *Z*. *Z*′ stands for the imaginary component, or reactance, and *Z*″ for the real part, or resistance. The complex number Z has both a real and an imaginary component. We used Equation (2) to calculate total impedance.

(1) $$Z=\sqrt{Z^{\prime 2}+Z^{\prime \prime 2}}$$

where *Z* is total capacity impedance, *Z*′ is the real part of the data collection, and Z″ is the imaginary part of the data collection. Inductors have positive reactance, whereas capacitors have negative reactance (imaginary part of the impedance). Although capacitive reactance is inversely proportional to electrical frequency, inductive reactance is proportional to electrical frequency. The magnitude of the electrical impedance with resistive and capacitive behavior can be estimated by considering [39]

(2) $$\left|Z\right|=R_{0}+\frac{R_{1}}{1+{\left(j\omega \tau \right)}^{\Psi }}$$

where $\tau =R_{1}C$, *C* is the capacitor in the system, $j=\sqrt{-1}$ is the imaginary part, ω is the angular frequency, $R_{0}$ and $R_{1}$ are resistors derived for an electrical model, and Ψ=1 describes an ideal capacitor.

### 2.4. Spectral Absorbance in C. albicans ATCC 10231 Biofilm

In order to conduct UV-vis spectroscopy studies, 96-well polystyrene plates (Nunc^®^ Thermo Scientific^®^, Waltham, MA, USA) were inoculated with 200 μL per well of yeast inoculum and incubated at 37 °C, allowing adherence of yeast. After incubation, the supernatant was removed, and a fresh RPMI 1640 medium was added. The studied plates were incubated until reaching 24 h of incubation. The UV-vis absorbance of yeast in vitro biofilm was measured in a multimode microplate reader (SpectraMax M3, Molecular Devices, CA, USA) in order to evaluate the optical absorption coefficient of the *C. albicans* sample. The sample was placed in the measurement device and was subjected to an absorbance spectrum studied with a wavelength ranging from 350 nm to 750 nm.

### 2.5. Studies of Ablation Effect over C. albicans ATCC 10231 Biofilms

Ablation effects in the samples were achieved using a Q-switched Nd:YAG laser system (Surelite II, Continuum, CA, USA) featuring 4 nanosecond pulse duration and emitting at 532 nm wavelength in a single-beam configuration. The focused beam diameter was around 2 mm with linear polarization. We propose to compare the effect of a single laser shot with 50 mJ compared to irradiating with 5 laser shots at 10 mJ each at 1 Hz. These conditions were systematically selected to obtain the comparative amount of incident energy to conduct nonlinear and linear ablation experiments.

**Figure 1:**
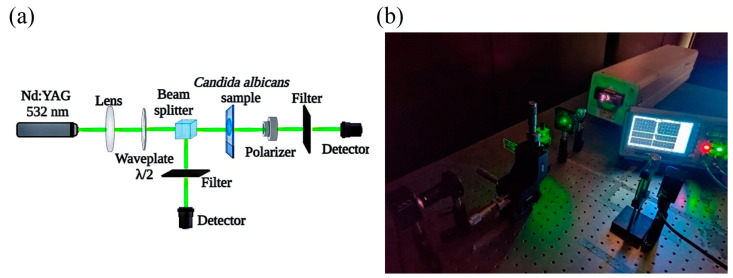
(**a**) Schematic illustration of the laser ablation experiment. (**b**) Representative photo of the experimental setup.

The laser energy can be absorbed by the yeasts, resulting in temperature elevation, vaporization, or melting of the sample. The inspection of the sample after the ablation threshold was conducted to determine the degree of fungal cell damage when the ablation procedure was finished. In order to examine how the laser irradiation modifies the *C. albicans* sample, we recorded optical micrographs. Figure 1 illustrates the experimental setup implemented in this study.

Figure 1 shows the experimental scheme of the system for laser ablation of the samples. A beam splitter (BS, Newport, Irvine, CA, USA) divides the primary coherent beam into two waves to measure the incident energy during the irradiation. Before reaching the sample, the incident beam goes through a focusing lens (KBX046AR.16 N-BK7 Bi-Convex Lens, Newport, USA) with a 25.4 mm effective focal length and a quarter-wave plate (05RP04-48 Newport, USA) to control the angle of polarization. The transmitted energy was measured by a detector (Power meter, 2936-R, Newport, USA).

When *C. albicans* ATCC 10231 is exposed to light, it absorbs some of the energy from the light and converts it into heat. This can cause an increase in the temperature of the sample. The density of *C. albicans* ATCC 10231 has been reported before [40], and the thermal conductivity can be significantly modified by its surroundings [41]. In order to carry out numerical simulations to describe the temperature induced by light in the sample, a mathematical approach was proposed to involve a fractional Caputo derivative, where α is a fractional coefficient. We conducted this approach with a numerical simulation with Equation (3) [42]

(3) $$T\left(t,a\right)=T_{m}+\left(T_{0}-T_{m}\right)e^{-\left(\frac{{K_{c}}^{h}}{a}t^{h}\right)}$$

where *T* is the temperature change in the sample, *T*_0_ is the temperature of the object’s surface (77 °C), $T_{m}$ is the temperature of the environment (25 °C), and *t* is the time that the sample was exposed. $K_{C}$ represents the cooling coefficient (0.20) and *h* is the fractional order (please see Supplementary Material for details).

The absorption coefficient ($a_{0}$) is related to the thickness and absorbance of the sample as described in Equation (4) [43].

(4) $$L=\frac{2.303A}{\alpha_{0}}$$

where A is the absorbance and 2.303 is the natural logarithm of 10; the estimated sample thickness L was 50 μm.

The nonlinear absorption coefficient was calculated by integrating the nonlinear absorption equation with the inherent characteristics of the materials. The equation to describe the nonlinear optical absorption behavior is [44]

(5) $$I_{A}=\frac{I_{0}\exp(-\alpha_{0}L)}{1+\beta_{I}I_{0}L_{eff}}$$

where $\beta_{I}$ is the nonlinear absorption coefficient, $I_{0}$ is the peak on-axis irradiance at focus, $\alpha_{0}$ as the absorption coefficient, L is the sample thickness, and $L_{eff}$ is the effective length $L_{eff}=\left[1-exp\left(-\alpha_{0}L\right)\right]/\alpha_{0}$.

While limiting harm to neighboring healthy cells by thermal transport induced by linear absorption at different wavelengths, the assistance of nonlinear optical phenomena may help to obtain a more uniform ablation effect [45]. Our main motivation in this study is to show that this innovative nonlinear strategy is feasible as a potentially effective treatment for *C. albicans* infections, with the goal of enhancing clinical results and minimizing unfavorable side effects.

## 3. Results and Discussion

**Figure 2:**
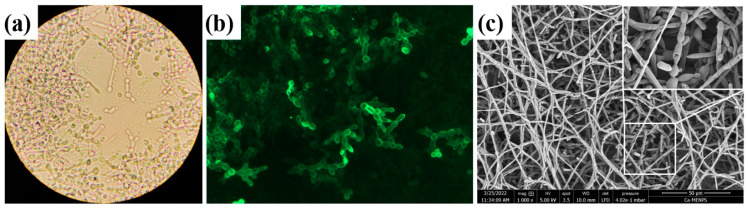
(**a**) Brightfield microscopy of *C. albicans* in vitro biofilm; (**b**) fungal cell wall α-D-mannosyl residues labeled with Concanavalin A stain, observed by epifluorescence microscopy; (**c**) biofilm architecture observed by scanning electron microscopy. In all cases, in vitro biofilm was started with an inoculum of 1 × 10^6^ blastoconidia/mL and was incubated at 37 °C for 24 h.

The morphology and architecture of yeast biofilm were studied, and the most representative results are illustrated in Figure 2. We observed a multilayered fungal growth with blastoconidia and pseudohypha proliferation, as can be seen in Figure 2a,c. Also, we showed the presence of mannose in the fungal cell wall by labeling it with the lectin Concanavalin A, as can be seen in Figure 2. The results of morphology exhibited by *C. albicans* ATCC 10231 depend on several factors, including the culture medium used, the growth conditions, and the length of time of growth.

In Figure 2a, it is possible to see how *C. albicans* extends in the analyzed region. Figure 2b shows the fungus using epifluorescence microscopy to observe the areas with the highest concentration of the sample. Figure 2c shows the SEM observations, in which the shape of the sample can be seen in detail with a closer view of the biofilm.

**Figure 3:**
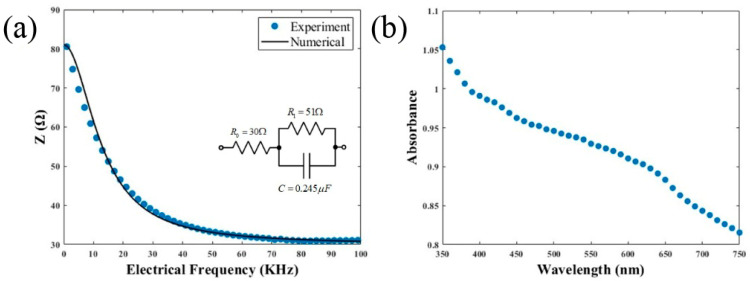
(**a**) Impedance vs. electrical frequency of *C. albicans* with 1 kHz to 100 kHz and 10 mV (**b**). Absorbance spectra of *C. albicans* ATCC 10231 in vitro biofilm starting with an inoculum of 1 × 10^6^ blastoconidia/mL and incubating at 37 °C for 24 h.

Electrical measurements of the sample can be associated with homogeneous yeast conditions and structure. Our findings demonstrated a capacitive behavior in *C. albicans* ATCC 10231 yeast, as plotted in Figure 3a. Figure 3b presents the UV-VIS absorbance of the samples with a monotonic decrease as a function of wavelength. The error bar of experimental data corresponds to ±1% in Figure 3a,b.

**Figure 4:**
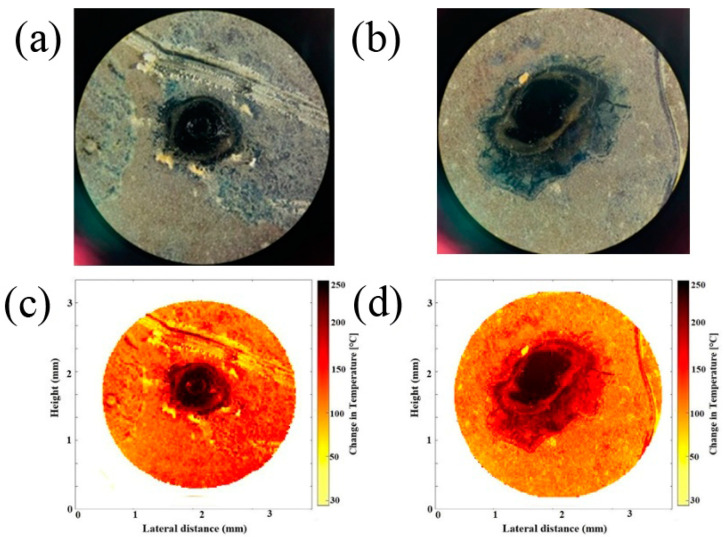
(**a**) Experimental results of optical ablation effect in *C. albicans* obtained by a single shot of a high-irradiance optical pulse. (**b**) Experimental results of optical ablation in *C. albicans* by a sequence of low-irradiance pulses. (**c**) Numerical results obtained by the Fractional Newton Cooling Law for ablation in the nonlinear optical effect. (**d**) Numerical results obtained by the Fractional Newton Cooling Law for ablation in the linear optical effect.

Figure 3a represents the electrical behavior of the sample as a function of electrical frequencies with an error bar of ±0.2%. Figure 4b shows the absorbance spectrum of the material. This capacitive behavior is crucial because it has direct consequences for electromagnetic energy transfer. This is because the capacitance behavior implies that charge and discharge can be more quickly as frequency rises.

We performed numerical fitting using a Cole–Cole model. It is an electrical model that can be used to calculate electrical impedance in biological samples [46]. In the context of *C. albicans*, electrical impedance analysis offers a non-invasive and quantitative way to assess the electrical properties of planktonic fungal cells and biofilms [47]. Fungal cells may be less susceptible to the damaging impacts of irradiation if their cell surface, including the cell membrane and cell wall, remains intact and structured. Therefore, we can take advantage of this capacitance behavior, which is critical for anticipating how cells would behave to various amounts of irradiance and is dependent on repetition rate and creating methods for *C. albicans* removal. The electrochemical impedance sensor has the potential for better sensitivity than blood culturing methods. Techniques for blood culturing may be impacted by several factors that influence the growth of *C. albicans* in a laboratory setting [48]. Another research approach that may be utilized is to examine how antibiotic and antifungal medications affect biofilms employing impedance analysis. The structure, composition, and other elements that could prevent the growth of the biofilm at the electrode contact were discovered to affect its impedance [49]. The interaction of resistive, capacitive, and inductive effects inside the sample is responsible for this behavior, which leads to conserving energy and redistribution events.

Figure 3b describes the optical absorbance that specifically allows us to predict the photoinduced behavior of *C. albicans* under optical irradiation at the wavelength selected for the ablation process, as has been suggested for fungal treatments [50].

In Figure 4, we show the photodamage experimental results. The estimated ablation threshold for the single shot measurement was close to 0.95 J/cm^2^. The degree of damage is determined by elements, such as laser wavelength, intensity, and time of exposure, as well as sample qualities [51]. These findings revealed that high-irradiance effects can be useful in generating more precise and symmetric photodamage. Descriptions for predictions of damage and energy transfer have been reported [52]. Fractional calculus seems to be mandatory to describe thermal transport in heterogeneous surfaces [53].

Optimization of optical parameters from the use of fractional calculus in laser irradiation studies seems to be attractive [54]. Additionally, fractional calculus may be utilized to comprehend how different biological structures respond to photodamage. When the ablation threshold is exceeded, the optical damage becomes irreversible in the fungus, causing an abrupt rupture in the cell walls. Two-photon absorption can lead to selective optical damage in the *C. albicans* sample using ultrashort laser pulses.

The photodamage experiment’s statistics can be described with an error limit of around 15%. Along with this, the incoming pulse optical energy varies by roughly 2.5%, which fits the error window offered by nonlinear phenomena. However, in the absence of optical damage, phononic processes account for most of the temperature change. The numerical information shown in Figure 4 considers the overall variation, a factor frequently employed in fractional calculations.

Fractional calculus provides a crucial tool for determining temperature and heat distribution in an inhomogeneous film [55]. Temperature changes within the irradiated area can be visualized by a single-shot laser and a series of high-intensity pulses. The black region in Figure 4c,d show the photodamage zones in the *C. albicans* sample. The temperature distributed in the film plane provides crucial information for understanding heat distribution on the sample surface. The simulation shows that the heat distributed on the surface of the biofilm is not uniform. While the results in Figure 4a stem from single-shot laser studies, the results in Figure 4b are derived from dynamic temperature evolution in a typical sample over a train of 5 nanosecond pulses at a repetition frequency of 1 Hz.

**Figure 5:**
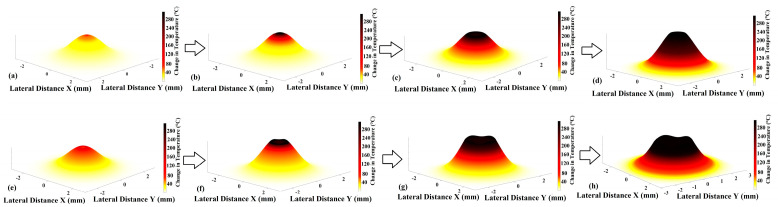
Numerical simulations estimated using FCL to describe a laser ablation process induced by (**a**) nonlinear optical effect with 1 pulse at 5 MW/cm^2^. (**b**) Nonlinear optical effect by 1 pulse at 7.5 MW/cm^2^. (**c**) Nonlinear optical effect by 1 pulse at 10 MW/cm^2^. (**d**) Nonlinear optical effect by 1 pulse at 12.5 MW/cm^2^. (**e**) Linear optical effect by 5 pulses at 1.25 MW/cm^2^. (**f**) Linear optical effect by 5 pulses at 1.875 MW/cm^2^. (**g**) Linear optical effect by 5 pulses at 2.5 MW/cm^2^. (**h**) Linear optical effect by 5 pulses at 3.125 MW/cm^2^.

In Figure 4a,b, it is possible to observe the different changes obtained after the sample is irradiated. Particular morphological changes can be seen in the sample, with Figure 4b being the one that shows the stronger modification in the irradiated area. This is due to the high-intensity laser pulses existing in the experimentation before the temperature propagates in a wide region of the biofilm. Using high-intensity laser pulses on the *C. albicans* sample, we can avoid damage in the surrounding of the irradiated zone. Optical ablation effects in a *C. albicans* ATCC 10231 biofilm can be assisted by a photosensitizer and light to produce reactive oxygen species that can kill microorganisms [56]. The laser light penetrates the biofilm and interacts with the cells to remove or reduce the size of the biofilm. As laser energy concentrates in the cell, molecules get excited and they vibrate at faster rates, causing the area’s temperature to rise. Increases in temperature can have a variety of effects on the behavior and structure of fungal cells. Figure 5 shows a numerical representation of the evolution of photodamage in the sample represented at different powers.

Figure 5a–d show the evolution of the nonlinear optical effect inducing ablation, while Figure 5e–h describe experiments with equivalent energy supplied in five pulses instead of just one. The ablated crater in the sample under nonlinear optical effects shows a conical shape, and the temperature distribution was higher than the other representations. It is important to mention that the temperature distribution has a prominent role in determining the overall behavior of the ablation process.

**Figure 6:**
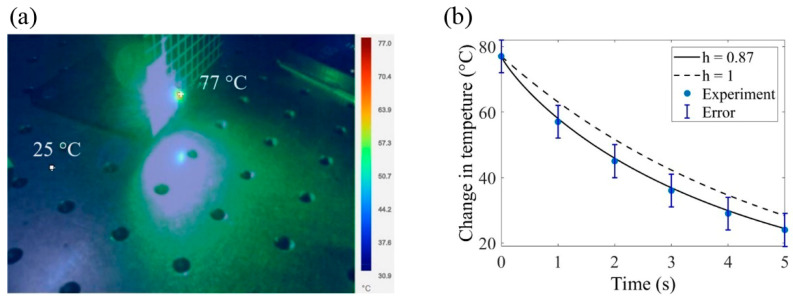
(**a**) Demonstration of the propagation of heat in *C. albicans* ATCC 10231 when the sample is exposed to an optical ablation. (**b**) Numerical simulation with α as the fractional order as 0.87, and dotted lines have α as 1 for temperature changes determined by *C. albicans* ATCC 10231 under normalized temperature vs. time.

One of the primary obstacles encountered throughout our research was that the laser ablation tests were limited to in vitro cultures of the *C. albicans* ATCC 10231, but this can be a base for future research. We use the Caputo fractional derivative to get around this restriction to describe the change in temperature in the samples studied. By considering the fractional order, we can better address the short-term memory effects and non-local interactions connected to thermal transfer in the sample. We can notice in Figure 6a that the hottest place in the sample was 77 °C, and the coldest zone in the sample was 25 °C. Figure 6b shows Caputo’s fractional calculus, which has a behavior that returns to its starting condition in less than 5 s, and we performed it in Equation (3).

In Figure 6a, experimental measurements that corroborate the temperature are recorded by a thermographic camera (Fluke). The maximum temperature reached in the sample was 77 °C, and it is represented as a small red dot where the sample is in Figure 6a, and the ambient temperature was 25°C. The error estimated in this experiment was ±5%. We used the fractional equation response of Newton’s cooling law for numerical simulations, as depicted in Figure 6b.

**Figure 7:**
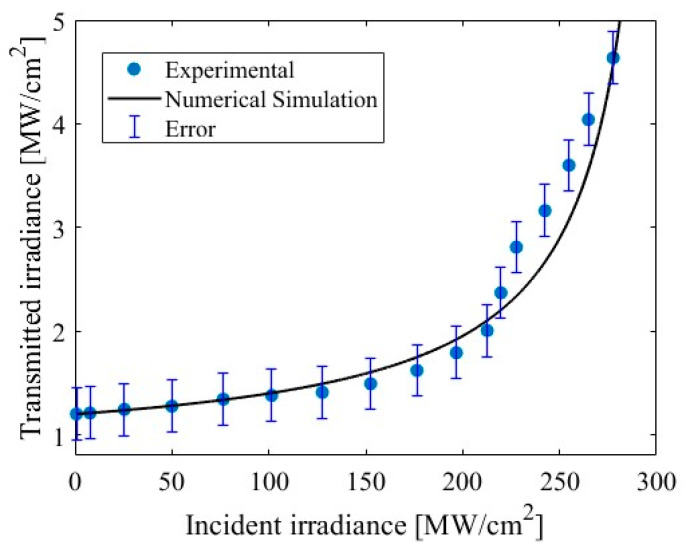
Transmitted irradiance vs. incident irradiance in *C. albicans*.

When a laser pulse hits the *C. albicans* biofilm, the cells of the wall slowly absorb the energy of the light, which causes local heat and damages the cell. It causes nonlinear effects, such as ionization and micromechanical destruction of irradiated regions. In the experiment, the temperature reached 77 °C. Immediately after ablation, the thermal release of the *C. albicans* image begins. However, the kinetics of this temperature do not represent the normal temperature dynamics. In terms of temperature, the *C. albicans* fungus has revealed that after a certain time, it reaches a maximum temperature, and then maintains it regardless of the time of exposure to the laser [57]. Furthermore, it has been demonstrated that by increasing the exposure time to 60 s to the optical ablation effect, there has been a strong decrease in the fungal strain [22]. This anomaly occurs due to memory effects due to temporal structural microdestruction caused by ultrafast laser ablation. Using the formalism of fractional calculation, we can adequately characterize the thermal evolution observed in a biological material. In order to guarantee the presence of nonlinear optical absorption, we measured the transmitted irradiance as a function of incident irradiance in the samples studied. The signature of a multiphotonic effect deriving in a saturated absorption effect with a $\beta =-2\times 10^{-6}$ cm/W was estimated, and the error bar is experimental data shown in Figure 7.

The ablation experiments were carried out in triplicate, and standard statistics were estimated. We compared our error bar by considering the evolution of the sample under irradiation. It is worth mentioning that quantitative data with a statistical approach should be considered to better describe the contribution of laser irradiation to the experimental data [11]. The intensity of light per unit area, or optical irradiance, is a key factor in numerous biological reactions. We employed Equation (5) to explain numerical simulation in Figure 7.

Fungal and yeast biofilms are a serious medical concern because they confer resistance to biotic and abiotic stress conditions in the microorganism [58], particularly due to their ability to resist common antibiotics [59]. However, promising research has explored the use of laser-based techniques, such as optical ablation and antimicrobial photodynamic inactivation, as potential treatments for biofilms [60]. The effectiveness of laser irradiation in suppressing the growth of the fungus has been reported in labeling antibacterial quantum dots, with complete ablation achieved at laser irradiance [53]. Antimicrobial photodynamic inactivation has been studied by utilizing a reactive oxygen species, leading to cell damage or death [61]. A reduction in *Candida* biofilm viability by irradiation has been reported by optical irradiation in direct incidence [62], and planktonic cultures influenced by sodium dodecyl sulfate irradiated by light reduce cell aggregation [63]. The photoinactivation of catalase seems to improve macrophage killing of intracellular *C. albicans*, confirming catalase as a significant biochemical target of blue light [64]. Furthermore, the impact of optical irradiation has shown a reduction in *C. albicans* levels according to strain testing [65].

## 4. Conclusions

Immediate applications for the use of nonlinear optics in inhomogeneous ablation effects in biological samples are proposed in this research. By measuring electro-capacitive behavior, fractional thermal transport, and multiphotonic absorption exhibited by *C. albicans* ATCC 10231, we analyzed photoenergy interactions with the potential for effective laser treatments in counters of fungi. Using high optical irradiance responsible for nonlinear optical phenomena, better control in the ablation zone can be obtained. The precise quantification of the temperature change caused by laser ablation employing fractional calculus provides important insights into the thermal dynamics of the treatment process studied. It highlights the use of high optical irradiance beyond limits in phononic propagation to optimize phototechnology functions assisted by ultrafast interactions.

## Data Availability

Data and materials are available upon reasonable request to C. Torres-Torres (ctorrest@ipn.mx).

## Supplementary Materials

The following supporting information can be downloaded at https://www.mdpi.com/article/10.3390/bioengineering11040333/s1, Supplementary Material S1: Fractional representation.
