# Supplementary material for: Multiphotonic Ablation and Electro-Capacitive Effects Exhibited by Candida albicans Biofilms
Source: Bioengineering (Basel). 2024 Mar 28;11(4):333. doi: 10.3390/bioengineering11040333 (PMC11048035; doi:10.3390/bioengineering11040333)
Supplement: Supplementary file 1 [file bioengineering-11-00333-s001.zip › bioengineering-2897180-Supplementary Material S1.pdf]

bioengineering

MDPI

Supplementary Material

Multiphotonic ablation and electro-capacitive effects exhibited by *Candida albicans* biofilms

Jose Alberto Arano-Martinez [1], José Alejandro Hernández-Benítez [2], Hilario Martines-Arano [3], Aída Verónica Rodríguez-Tovar [2], Martín Trejo-Valdez [4], Blanca Estela García-Pérez [2] and Carlos Torres-Torres [1,*]

[1] Sección de Estudios de Posgrado e Investigación, Escuela Superior de Ingeniería Mecánica y Eléctrica Unidad Zacatenco, Instituto Politécnico Nacional, Ciudad de México, 07738, Mexico;
[2] Departamento de Microbiología, Escuela Nacional de Ciencias Biológicas, Instituto Politécnico Nacional, Ciudad de México, 11340, Mexico
[3] Escuela Superior de Ingeniería Química e Industrias Extractivas, Instituto Politécnico Nacional, Ciudad de México, 07738, Mexico
* Correspondence: ctorrest@ipn.mx

Fractional representation

Optical nonlinearities are intimately connected to optical phase modification. The dynamic photothermal energy transfer appears to be responsible for the modulation of optical pulses in nanoparticles. We computed the thermal transport of energy in the sample under the effect of laser pulses to explain the evolution of the optical phase in our tests. The temperature reaction of the sample under the impact of laser pulses is mathematically represented by [30]:

$$\frac{\partial T(z,t)}{\partial t} = K\frac{\partial^2 T(z,t)}{\partial z^2} + Q(z,t) \tag{S1}$$

If x is a radial length differential, K represents thermal diffusivity, $K = K_0/C$; where K_0 is thermal conductivity, is density, C is specific heat, and Q is laser heat. The Fourier series used to describe laser pumping was defined as follows [30]:

$$f(t) = W_\alpha\left[\frac{t_0}{\tau} + \sum_{n=1}^{\infty} sen\left(\frac{n\pi t_0}{\tau}\right)\cos\left(\frac{2n\pi}{\tau}t\right)\right] \tag{S2}$$

When t_0 is the pulse width, is the period, and W_0 represents the optical energy absorbed by the sample, with $W_\alpha = \gamma$, where is an absorptive constant, γ is the optical irradiance, and n is the optical Kerr effect. From equation (S1), various accumulations of thermal energy under different irradiance circumstances may be predicted, as well as variable cooling periods due to thermal transport in the samples. The Newton cooling law may be used to better describe the phase change exhibited in [30]:

$$\frac{dQ_e}{dt} = -k_c\left(T_0 - T_m\right) \tag{S3}$$

K_c is the cooling coefficient, and $K_c = Q_e H$. Here, Q_e is the thermal energy, H is the heat transfer coefficient, T_0 is the surface temperature of the item, and T_m is the ambient temperature. Because this rule gives idealized conditions that do not meet the real response, the solution of the integer order for the classical Newton cooling equation does not fit the experimental data. However, the fractional computation provides for an answer that is extremely as the actual behavior.

Although the response of Newton's classical cooling equation was not adjusted to the measured values, it was recommended to evaluate equation (S1) in a different method, employing the fractional derivative of Caputo to produce a better fitting. The Newton cooling equation is composed of the following terms [30]:

$$T(t,\alpha) = T_m + (T_0 - T_m)\,\mathrm{e}^{-\left(\frac{K_c{}^{\alpha}}{\alpha} t^{\alpha}\right)} \qquad \text{(S4)}$$

Equation S4 is the one shown above and is the one used to obtain the Caputo fractional change in temperature represented in Figure 5b for numerical simulation.
